# Prison Labour

**Published:** 1896-05-16

**Authors:** 


### Modern Sociology.

### PRISON LABOUR.

In the warrant appointing the Departmental Committee on Prisons, which issued its report on April 10th, 1895, the Home Secretary directs the Committee, amongst other things, to inquire into prison labour and occupation, with special reference to the moral and physical condition of the prisoners. In their report (par. 47, &c.) the Committee say: "In the consideration of this question (unproductive labour) we start from the principle that prison treatment should have as its primary and concurrent objects deterrence and reformation." The report goes on to consider in the succeeding paragraphs productive labour, the nature and extent of prison industries, male and female prisoners' work, the quality of prison work, the extension and organisation of prison industries and prison occupation generally. At the conclusion of the report there is a summary of the principal recommendations of the Committee, three paragraphs of which deal with the question of prison labour. Par. v. recommends "that prisoners should not forfeit marks by reason of physical or mental weakness or illness. Power to earn remission of sentences should be extended to local prisons." Par. vi., "That unproductive labour should be abolished wherever possible. Association for productive work and technical instruction to be extended gradually, and with due caution, throughout the prisons. Productive prison industries to be increased as much as possible, especially as regards gardening, farming, and land reclamation. Every effort is to be made to secure additional orders from Government departments. Prisoners to be enabled to earn something continuously during their sentence, provided that the money so earned is given to them through the Prisoners' Aid Society." Par. vii. "recommends that the official in the Prisons Department who supervises the prison industries should have a higher degree of responsibility and authority. The number of skilled teachers of industries in the prison service to be increased, and extra allowances to be granted to warders who qualify themselves for the supervision of work requiring a certain amount of skill. In each prison an official to hold a position somewhat similar to that of 'manufacturer' prior to 1878 for the purpose of directing the prison industries." The hearing of evidence occupied the Committee twenty-four days, and 11,815 questions were asked and answered, so that it will be readily understood that the Committee went very thoroughly into their inquiry. The above extracts show their recommendations on the subject of prison labour. In the appendix is a most interesting description of the prison systems of foreign countries. An important addendum is a short description of the Elmira Reformatory, New York, the object of which is, as far as practicable, to place the prisoner in "conditions parallel with those of free life," there to work out his institutional career. In all countries, both in Europe and the United States, the principle of reformation appears to be universally acknowledged to hold the chief place in the prison system, and its application has been abundantly justified by the results attained.

There are two kinds of prison labour, unproductive and productive, each of which may again be subdivided into "cellular and associated." By the present prison system in England for the first nine months of their sentences convicts are, whether in local or convict prisons, kept in isolated confinement. After this period they are put to "associated" labour on public works, land reclaiming, &c. What is known as "first class" hard labour to which prisoners are subjected for the first month of their sentence consists of the treadmill, shot-drill, stone-breaking, and other similar mechanical punishments. This class of labour has been almost universally condemned by the highest authorities on prisons as being irritating, depressing, and debasing to the mental faculties; brutalising in its effects and keeping the mind in a state of mental vacuity. One of the chief difficulties to be contended with is that at present local prisons are generally situated in the heart of large towns, and work has to be provided for the prisoners within their walls. As to the respective advantages of "cellular and associated" labour, the chief fear of the later form has hitherto been the fear of "contamination." But in the opinion of the best authorities, this can be obviated by making careful selections of prisoners for "associated" work. One great difficulty hitherto experienced has been to find a sufficiency of suitable work, especially for male prisoners, and this has been increased by the shortness of the great majority of the sentences, which renders it almost impossible to train short-sentence men to any skilled trade. This difficulty has been further aggravated by the outcry in the House of Commons, and outside of it, against the competition of prison with free labour. But let it be remembered that the value of prison labour, including the domestic services of the prisons, is estimated at from £110,000 to £120,000 per annum; and that, admitting that even the ordinary prisoner is equally efficient with a free workman, which is certainly not the case, it is calculated that the abolition of unproductive labour, and the employment of all now so engaged upon productive labour, would only increase the interference of prison with outside labour in the proportion of 1 to 2,500. When these facts and figures are taken into account, it will be seen that the opposition to productive labour in prisons has no firm basis to rest upon. As to the work of female prisoners, laundry and domestic work is chiefly given to them, and for cases of isolated confinement knitting and needlework of all kinds has been found most beneficial. It has been suggested that a large amount of "washing" now done for Government in London might be done by female prisoners and by the inmates of rescue homes, to the amount of from £3,000 to £4,000 per annum. In France a great deal of printing is done for the Government by prisoners. The best authorities are all agreed that outdoor work, such as the reclamation of land and gardening, is most beneficial in its effects, being healthy, productive, varied, and of a more or less interesting character.

